# P-1347. Characterizing Atypical Antibiotic Non-Susceptibility Features of Emergent U.S. Carbapenem-resistant *Acinetobacter baumannii* Lineages

**DOI:** 10.1093/ofid/ofae631.1524

**Published:** 2025-01-29

**Authors:** Emma Graffice, Md Hasanul Banna Siam, Juan Calix, Megan Amerson-Brown

**Affiliations:** University of Alabama in Birmingham Heersink School of Medicine, Birmingham, Alabama; University of Alabama in Birmingham Heersink School of Medicine, Birmingham, Alabama; University of Alabama in Birmingham Heersink School of Medicine, Birmingham, Alabama; University of Alabama in Birmingham Heersink School of Medicine, Birmingham, Alabama

## Abstract

**Background:**

Due to elevated genetic divergence between different Carbapenem-resistant (CR) *A. baumannii* (Ab) lineages, CR*Ab* isolates belonging to clonal groups (CGs) currently emerging in the U.S. may display traits that differ from those of unrelated international CGs. Variations in CG-associated physiology could especially affect the choice and interpretation of antimicrobial susceptibility tests (AST) for these multidrug resistant lineages.

**Methods:**

We integrated multimodal AST and comparative genomic analysis of CG108, CG406, and CG499 isolates identified in two U.S. cities since 2017. Lineage-specific resistance features were queried among genomes of isolates from other U.S. locations. We further investigated a unique sulfonamide (SXT) heteroresistance trait via mutational analysis.

**Results:**

Most antibiotic non-susceptibility was attributable to conventional, lineage-independent resistance determinants on mobile genetic elements. In contrast, a FtsI A515V polymorphism shared among CG108 genomes appears to confer carbapenem resistance independent of carbapenamases, while genomes of various CG406 CR*Ab* isolates lacked any readily identifiable CR determinant. We observed antibiotic-specific heteroresistance against sulbactam (SAM) and imipenem among CG108 isolates and against SXT among CG499 isolates. Integrity of the *wzc* gene in a CG499 isolate was necessary for high-molecular weight capsule polysaccharide (KPS) production and SXT heteroresistance. Despite lacking any conventional resistance determinants, the *wzc-*deficient mutant and all other CG499 isolates displayed greater resistance to SXT compared to most susceptible *Ab* isolates.

**Conclusion:**

We describe AST features unique to emerging CR*Ab* CGs and independent of conventional extrinsic resistance genes. CG499 SXT heteroresistance appears to rely on the ability to produce high-molecular weight KPS, though another unidentified mechanism also contributes to decreased susceptibility compared to wild-type. We propose ongoing surveillance efforts must recognize lineage-related variations to best tailor new strategies against the evolving nature of CR*Ab* disease.

**Disclosures:**

**Megan Amerson-Brown, PhD**, Selux diagnostics: Grant/Research Support

